# Total rupture of Achilles tendon induces inflammatory response and glial activation on the spinal cord of mice

**DOI:** 10.1590/1414-431X2023e12391

**Published:** 2023-10-13

**Authors:** D.R. De Paula, M.S. França, L.K.R. Leão, A.A. Maciel, T.A.A. Moura, S.A.S. de Moraes, C.P. Bahia, R.S. Borges, E.J.O. Batista, A.C.F. Passos, K.R.H.M. Oliveira, A.M. Herculano

**Affiliations:** 1 Instituto de Ciências Biológicas, Universidade Federal do Pará, Belém, PA, Brasil; 2 Instituto de Ciências da Saúde, Universidade Federal do Pará, Belém, PA, Brasil; 3 Núcleo de Medicina Tropical, Universidade Federal do Pará, Belém, PA, Brasil

**Keywords:** Glia, Nitric oxide, Tendinopathy, Spinal cord

## Abstract

Rupture of Achilles tendon is a common accident affecting professional and recreational athletes. Acute and chronic pain are symptoms commonly observed in patients with rupture. However, few studies have investigated whether Achilles tendon rupture is able to promote disorders in the central nervous system (CNS). Therefore, the current study aimed to evaluate nociceptive alterations and inflammatory response in the L5 lumbar segment of Balb/c mice spinal cord after Achilles tendon rupture. We found increased algesia in the paw of the ruptured group on the 7th and 14th days post-tenotomy compared with the control group. This phenomenon was accompanied by overexpression of cyclooxygenase-2 (COX-2) and inducible nitric oxide synthase-2 (NOS-2) as well as hyperactivation of astrocytes and microglia in nociceptive areas of L5 spinal cord as evidenced by intense GFAP and IBA-1 immunostaining, respectively. Biochemical studies also demonstrated increased levels of nitrite in the L5 spinal cord of tenotomized animals compared with the control group. Thus, we have demonstrated for the first time that total rupture of the Achilles tendon induced inflammatory response and nitrergic and glial activation in the CNS in the L5 spinal cord region.

## Introduction

Rupture of Achilles tendon is a common injury observed in recreational and professional athletes (1-3). Partial or total rupture of the Achilles tendon demands special attention since prolonged periods of tissue impairment can induce permanent alterations in normal gait pattern of patients (4). The treatment of a ruptured Achilles tendon usually involves surgical intervention, and recovery time varies among individuals (5,6). As widely described in the literature, the time elapsed from tendon rupture to surgical repair is predictive of Achilles tendon recovery (7). Different studies show that localized pain and changes in gait pattern are symptoms commonly described in injured subjects (8,9). In fact, the biochemical and histological alterations in the area of the ruptured Achilles tendon are well documented, but there are few studies describing the impact of this injury on the central nervous system (CNS) (10-12).

The Achilles tendon is connected to the gastrocnemius and soleus muscles (13), and under normal conditions, the tendon tissue is poorly innervated. In fact, anatomical and histological studies show that the tendon is located in an adjacent structure (paratenon) that forms nerve plexuses, from which small branches penetrate into the tendon sheath (epitenon) (12-17). Notably, in pathological conditions there is intense nerve fiber growth (sensory and autonomic fibers) that inserts into collagen fibers. This phenomenon is closely associated to regulation of pain, inflammation, and tissue repair (14-16).

Neuronal cells that process information from the Achilles tendon are localized in the lumbar intumescence of the L5 segment (18). Few studies have evaluated how the spinal cord responds to injury in the Achilles tendon (19,20,21). Furthermore, it remains unclear if Achilles tendon injuries are able to trigger an inflammatory response in the spinal cord of injured patients. As previously demonstrated, the inflammatory response in injured spinal cord can be evidenced by the activation of astrocytes or neurons (22,23) and by the expression of inflammatory enzymatic mediators such cyclooxygenase-2 (COX-2) and inducible nitric oxide synthase-2 (NOS-2). Astrocytes and activated microglia produce inflammatory mediators such as prostaglandins and nitric oxide (NO), which are important indicators of CNS injury (24,25). The production of these inflammatory agents is frequently evaluated by local expression of NOS-2 and COX-2, which are the enzymes responsible for their synthesis (24,26).

The current study aimed to describe the effect of Achilles tendon rupture on paw mechanical sensitivity and on spinal inflammatory response of tenotomized mice utilizing biochemical and histological evaluation.

## Material and Methods

### Animals and experimental design

A total of 39 Balb/c mice (males, 6-8 weeks old, weighing 25-30 g) were provided by the animal facilities of the Federal University of Pará (UFPA) and kept in polypropylene cages at 25°C under a controlled dark/light cycle (12:12 h) with food and water *ad libitum*. Animals were anesthetized by intraperitoneal injection of ketamine/xylazine solution (90/5 mg/kg). The tibia region of the right paw was manually trichotomized and a longitudinal skin incision (about 0.5 cm) was made to access the Achilles tendon. This step was followed by the complete transversal section of the tendon as previously described (12). The animals (n=6 per group) were separated into control group (CG), which was not submitted to the surgical procedure, and ruptured group (RG), which had the Achilles tendon transected at 0.5 cm from its calcaneal insertion. These steps were followed by local asepsis and skin suture using a 4.0 nylon monofilament. The experimental animals were returned to their cages without movement restriction. The spinal cord of control and ruptured animals were collected on the 7th (n= 9 per group) and 14th (n= 9 per group) days post-surgery after tissue fixation by transcardiac perfusion with saline solution (0.9%) and 4% paraformaldehyde (PFA). The lumbar region of the spinal cord was removed by laminectomy and was then post-fixed with 4% PFA for 24 h.

The samples were cryoprotected by sequential immersion in a gradient of sucrose solution (10, 20, and 30%). Then, the specimens were soaked in Tissue Tek^®^ (Sakura Finetek, Inc., USA). Afterwards, the L5 level was identified and sectioned at 10 µm utilizing a Leica cryostat set to -24°C (model CM3050, Germany). Immunohistochemical (9 animals, n=3 per group) and biochemical (18 animals, n=6 per group) analyses were conducted. The animal experiments were handled in strict compliance with the guidelines of Brazilian law No. 11.794/2008 for the care and use of animals for scientific purposes. The protocol was previously approved by the Ethical Committee for Care and Use of Laboratory Animals (CEUA) from UFPA (Protocol number 8179020318).

### von Frey behavioral test (mechanical sensitivity)

**Figure 1:**
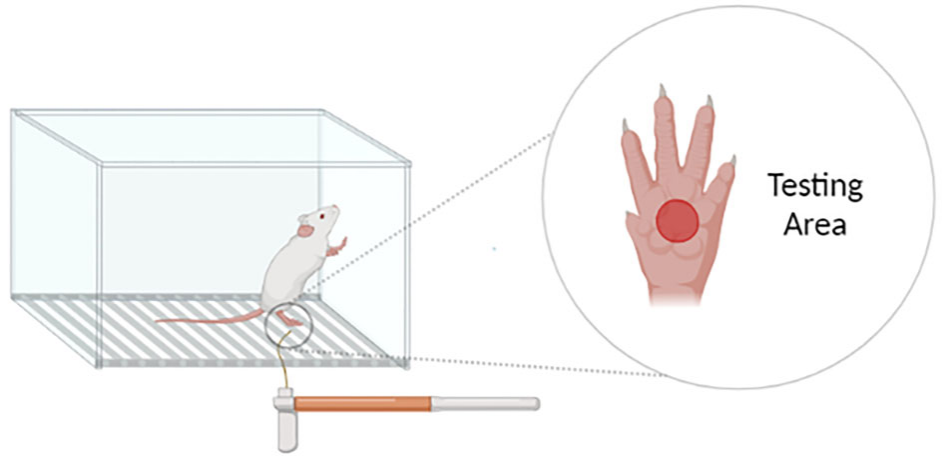
Image of the von Frey apparatus. The von Frey filaments are inserted through the elevated mesh platform to press the paw of the mouse. The animal is repeatedly stimulated with increasingly strong filaments to determine the threshold at which the paw withdrawal response is reliably elicited.

The behavioral analysis was carried out with 12 animals (n=4 per group). The hind paw withdrawal threshold was determined using von Frey hairs ranging from 0.02 to 10 g. All experimental procedures were performed blindly, such that the experimenter was not aware of the group (control or ruptured mice) that were being tested. The protocol used in the current study was made in accordance with Chaplan et al. (27), with few variations. The tests for control and ruptured groups began after 5 min of habituation. The series of von Frey hairs was applied from below, in a customized platform as shown in Figure 1. The ipsilateral hind paw of control or ruptured mice were pressed with filaments of increasing stiffness (0.02-10 g) applied to the plantar surface for 5-6 s. Each filament was applied 10 times and the minimum value that caused at least 3 responses was recorded as paw withdrawal thresholds (PWT). Acute withdrawal, biting, licking, or shaking of the ipsilateral posterior limb and vocalization were considered positive signs of withdrawal. The average of these values was used for data analysis. The withdrawal threshold was determined in each animal before surgery and 7 and 14 days after surgery in independent groups.

### Immunostaining assays

Slices of L5 spinal cord were washed twice with pH 7.2 phosphate buffer (PBS) for 30 min and incubated in ammonia chloride solution (pH 8.0) for 40 min. The tissues were permeabilized with Triton X-100 and 3% BSA solution at room temperature for 1 h. The samples were incubated for 20 min in 3% hydrogen peroxide in order to inhibit endogenous peroxidases. This step was followed by overnight incubation with primary anti-GFAP (H-50, Santa Cruz Biotechnology, USA, 1:200), anti-Iba-1 (ab5076, Abcam, USA, 1:200), anti-NOS-2 (Santa Cruz Biotechnology, 1:200), and anti-COX-2 (SAB42, Sigma-Aldrich, USA, 1:200) at 4°C and posterior washes with pH 7.2 PBS. The slices used to evaluate GFAP and IBA-1 expression were incubated for 2 h with secondary antibody goat anti-rabbit IgG-PE (sc-3739, Santa Cruz Biotechnology, 1:1000) and Alexa Fluor 488 anti-goat ab150129 (ab150129, Molecular Probes, USA, 1:1000), respectively, both conjugated with peroxidase and revealed using DAB peroxidase substrate (34002, ThermoFisher, USA). NOS-2 and COX-2 expression were evaluated by immunofluorescence after incubation for 2 h with secondary anti-body Alexa Fluor 488 or Alexa Fluor 594 (Santa Cruz Biotechnology, 1:1000) at room temperature. Cell nuclei were stained with DAPI probe (d9542, Sigma-Aldrich, 1:1,000). Finally, tissue sections were washed and mounted on lamina containing N-propyl-gallate and then visualized by fluorescence microscopy (Zeiss Mod, Germany). Glial cell reactivity quantification (GFAP and IBA-1) was performed by manual counting, using 4 images of each field (1.530 mm^2^) of the L5 spinal cord segment on ImageJ^®^ software (USA). The spinal cord was labeled, and the number of cells stained with GFAP and IBA-1 in the dorsal and ventral horns as well as in the ipsilateral and contralateral sides of the lesion was determined (28). Data are reported as means±SD.

### Biochemical analysis

Nitric oxide production in the L5 spinal cord was measured as previously described by Darmani et al. (29). Control (n=6) and ruptured mice (n=6) had their lumbar spinal cord dissected at L5 level as described above. The tissues were homogenized in saline solution and then 700 μL of the solution was centrifuged (174 *g*, 10 min, 25°C), and the supernatant was collected. After that, 500 μL of the sample was mixed with the same volume of Griess reagent (1% sulfanilamide, 0.1% naphthalene, and 5% phosphoric acid, Sigma-Aldrich). Absorbance values were read at 540 nm and referred to the nitrite standard curve. Nitrite levels in the samples were normalized by the concentration of protein found, and protein content was measured by the Bradford method.

### Statistical analysis

The appropriate statistical tests were selected after using Kolmogorov-Smirnov normality test. Data are reported as means±SD and the difference between control and ruptured groups was evaluated using one-way ANOVA followed by Tukey *post hoc* tests. For behavioral data, two-way ANOVA followed by Tukey *post hoc* was used. Statistical analyses were made with BioStat 5.0 (USA) and P≤0.05 was considered as significant.

## Results

### Rupture of Achilles tendon potentiated algesia response in mice paws

**Figure 2:**
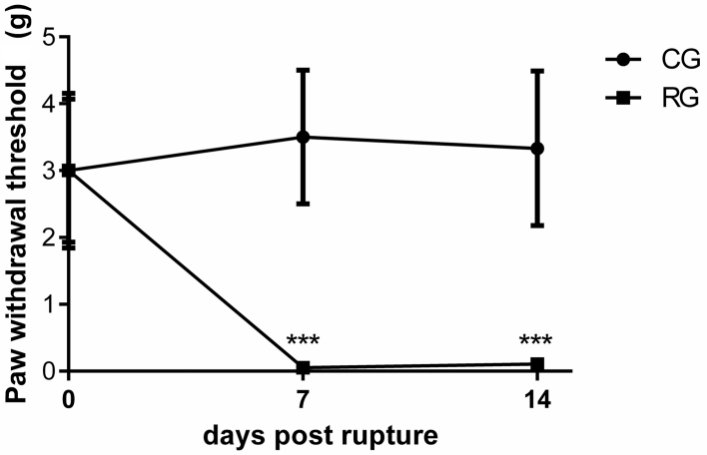
Effects of Achilles tendon rupture in the mechanical nociceptive threshold. CG (control group) and RG (rupture group) were analyzed before surgery and 7 and 14 days post-Achilles tendon tenotomy. Data are reported as means±SD. ***P<0.01 CG *vs* RG, n=6/group (two-way ANOVA followed by Tukey’s *post hoc* comparisons).

As demonstrated in Figure 2, animals submitted to total tenotomy of Achilles tendon showed a significant decrease in PWT values for the ipsilateral paw (0.05±0.18 g) compared with control group (3.5±1.0 g) on the 7th day post-lesion. Similar results were observed on the 14th day post-lesion, with the ruptured group showing lower values of PWT (0.1±0.07 g) than the control group (3.3±1.1 g). The baseline test showed no significant difference among the groups.

### Effect of Achilles tendon rupture on the microglial activation in the L5 spinal cord segment

**Figure 3:**
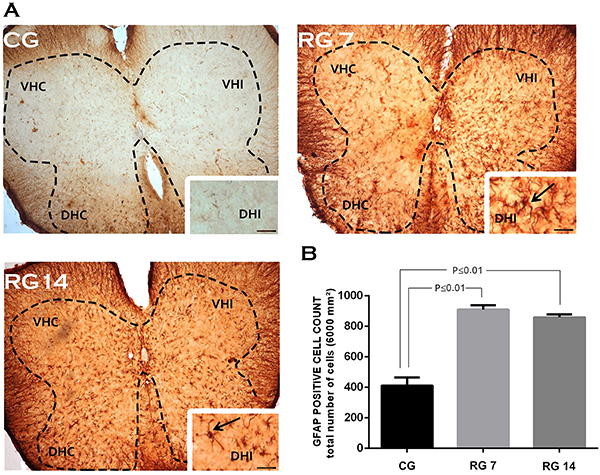
Effects of Achilles tendon rupture on astrocyte reactivity in the spinal cord. **A**, GFAP immunostaining of transversal sections of mice spinal cord (segment L5) on the 7th and 14th days post-Achilles tendon rupture. **B**, GFAP-positive cell count in the L5 spinal cord of the groups. Data are reported as means±SD, n=6/group (one-way ANOVA followed by Tukey's *post hoc* comparisons). VHC: ventral horn contralateral; VHI: ventral horn ipsilateral; DHC: dorsal horn contralateral; DHI: dorsal horn ipsilateral. Black arrows in the enlarged images show strong astrogliosis in the dorsal horn of the ipsilateral side to injury (scale bar=100 μm). CG: control group; RG 7: rupture group analyzed on the 7th day after tenotomy; RG 14: rupture group analyzed on the 14th day after tenotomy.

**Figure 4:**
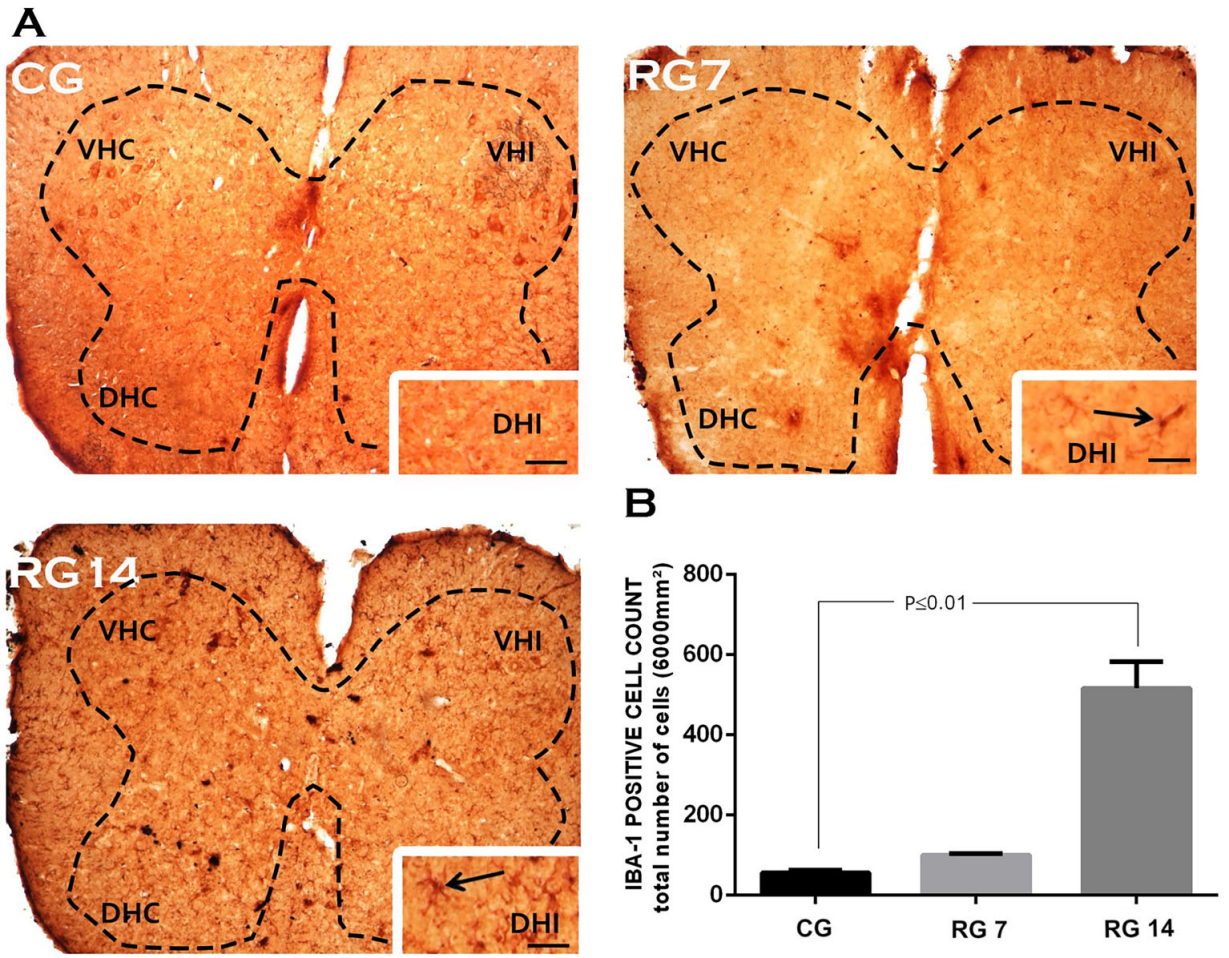
Effects of Achilles tendon rupture on microglial reactivity in the spinal cord. **A**, IBA-1 immunostaining of transversal sections of mice spinal cord (segment L5) on the 7th and 14th days post-Achilles tendon rupture. **B**, IBA-1-positive cell count in L5 spinal cord. Data are reported as means±SD, n=6/group (one-way ANOVA followed by Tukey's *post hoc* comparisons). VHC: ventral horn contralateral; VHI: ventral horn ipsilateral; DHC: dorsal horn contralateral; DHI: dorsal horn ipsilateral. Black arrows in the enlarged images show strong microgliosis in the dorsal horn of ipsilateral side to injury, mainly in RG 14. Scale bar=100 μm. CG: control group; RG 7: rupture group analyzed on the 7th day after tenotomy; RG 14: rupture group analyzed on the 14th day after tenotomy.

Immunolabeling analysis in L5 spinal cords showed significant staining for GFAP protein in the control group (Figure 3). This phenomenon can be seen in Figure 3A and B, which also demonstrate that ruptured animals showed an increased number of GFAP-positive cells on the 7th (n=910±29 cells/µm^2^) and 14th (n=860±20 cells/µm^2^) days post-surgery compared with the control group (n=412±54 cells/µm^2^). In addition, our data showed that Achilles tendon rupture induced microglial activation in the L5 spinal cord. As described in Figure 4, the control group had fewer IBA-1-positive cells (n=60±7 cells/µm^2^) compared to tenotomized animals in the L5 spinal cord on the 7th (n=100±2 cells/µm^2^) and 14th days post-surgery (n=500±70 cells/µm^2^). Spinal cord glial reactivity was predominant on the ipsilateral side to tendon rupture and concentrated in the dorsal horns.

**Figure 5:**
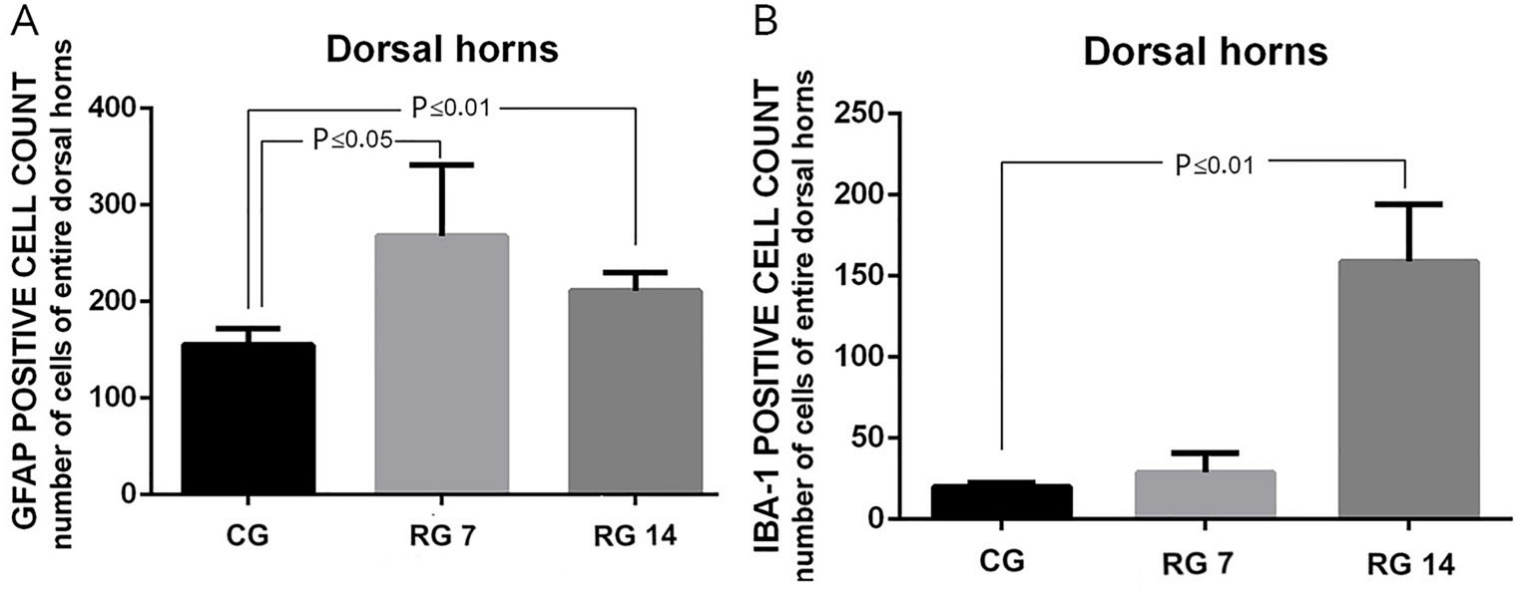
Effects of Achilles tendon rupture on glial reactivity in dorsal horns. **A**, GFAP-positive cell count in dorsal horns of L5 spinal cord. **B**, IBA-1-positive cell count in dorsal horns of L5 spinal cord. Data are reported as means±SD, n=6/group assessed (ANOVA followed by Tukey's *post hoc* comparisons). CG: control group; RG 7: rupture group analyzed on the 7th day after tenotomy; RG 14: rupture group analyzed on the 14th day after tenotomy.

**Figure 6:**
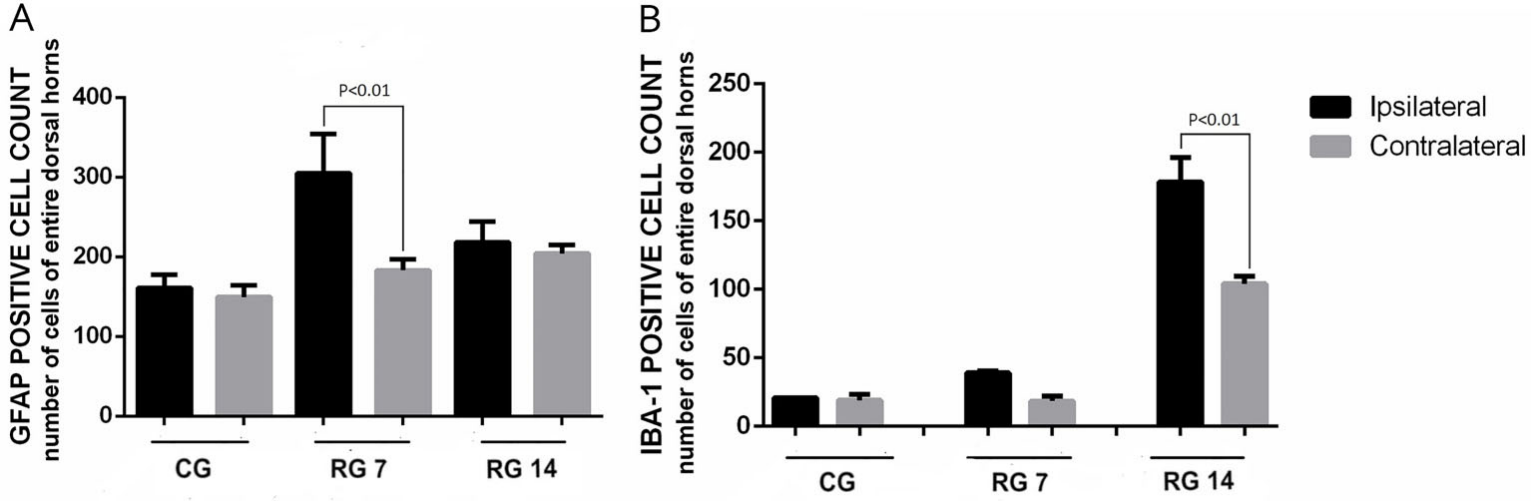
Effects of Achilles tendon rupture on glial reactivity between the ipsi- and contralateral sides. **A**, GFAP-positive cell count in dorsal horns of L5 spinal cord. **B**, IBA-1-positive cell count of only dorsal horns of L5 spinal cord. Data are reported as means±SD, n=6/group (one-way ANOVA followed by Tukey's *post hoc* comparisons). CG: control group; RG 7: rupture group analyzed on the 7th day after tenotomy; RG 14: rupture group analyzed 14th day after tenotomy.

As shown in Figure 5A, tendon rupture induced an increase in the number of GFAP-positive cells on the 7th (267±73 cells/µm^2^) and 14th (211±18 cells/µm^2^) days post-rupture compared with non-ruptured animals (155±16 cells/µm^2^). On the other hand, as shown in Figure 5B, a higher number of IBA-1-positive cells was observed only on the 14th day post-rupture (control=20±2.7 cells/µm^2^ *vs* ruptured group=158.75±35.5 cells/µm^2^). The ipsilateral side of the rupture presented an increased number of GFAP-positive cells 7 days post-surgery compared with the contralateral side of the lesion (ipsilateral=305±49.4 *vs* contralateral=183±14.1 cell/µm^2^), and no significant difference was observed 14 days post-surgery (Figure 6A). On the other hand, rupture of Achilles tendon did not alter the number of IBA-1 positive cells 7 days post-surgery, but 14 days post-surgery, these values were significantly increased compared with the contralateral side (ipsilateral=178.5±17.6 *vs* contralateral=104±5.6 cell/µm^2^) (Figure 6B).

### Inflammatory activation in L5 spinal cord induced by tendon rupture

**Figure 7:**
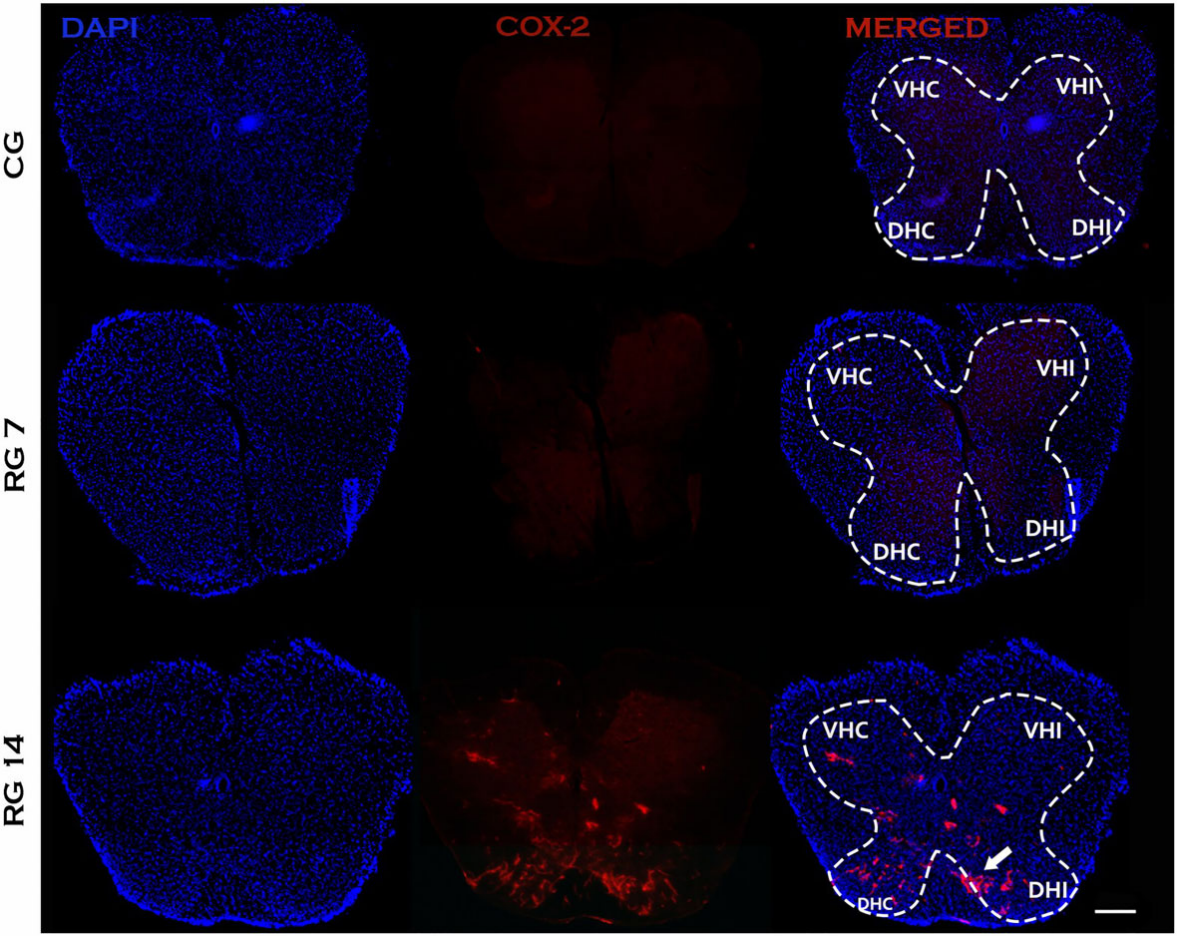
Inflammatory response of the spinal cord induced by Achilles tendon rupture. Immunofluorescence to COX-2 of transversal sections of mice spinal cord (segment L5) on the 7th and 14th days post-Achilles tendon rupture. The nuclei of cells were stained with DAPI. VHC: ventral horn contralateral; VHI: ventral horn ipsilateral; DHC: dorsal horn contralateral; DHI: dorsal horn ipsilateral. The white arrow shows strong reactivity in the dorsal horn of ipsilateral side to injury. n=6/group. Scale bar=100 μm. CG: control group; RG 7: rupture group analyzed on the 7th day after tenotomy; RG 14: rupture group analyzed 14th day after tenotomy.

**Figure 8:**
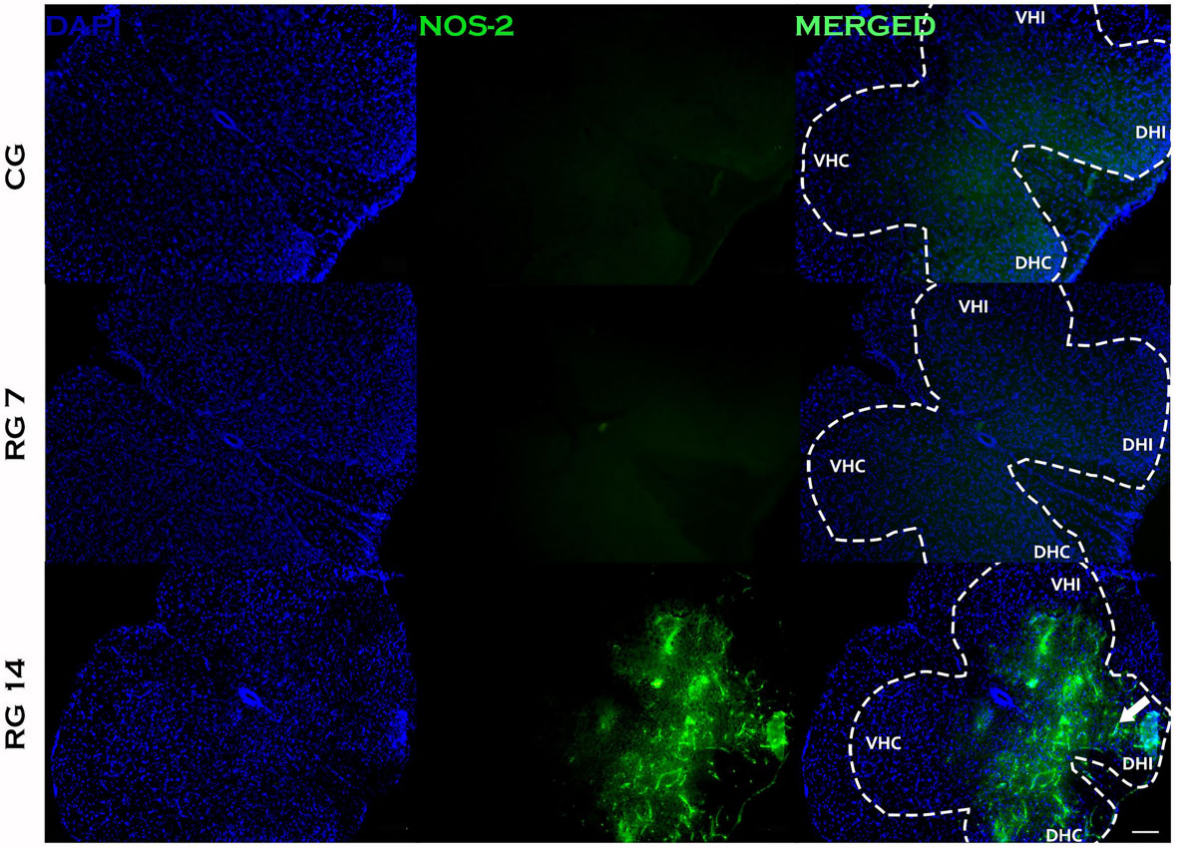
Inflammatory response of the spinal cord induced by Achilles tendon rupture. Immunofluorescence to NOS-2 of transversal sections of mice spinal cord (segment L5) on the 7th and 14th days post-Achilles tendon rupture. The nuclei of cells were stained with DAPI. VHC: ventral horn contralateral; VHI: ventral horn ipsilateral; DHC: dorsal horn contralateral; DHI: dorsal horn ipsilateral. White arrow shows strong reactivity in the dorsal horn of ipsilateral side to injury. n=6/group. Scale bar=100 μm. CG: control group; RG 7: rupture group analyzed on the 7th day after tenotomy; RG 14: rupture group analyzed 14th day after tenotomy.

**Figure 9:**
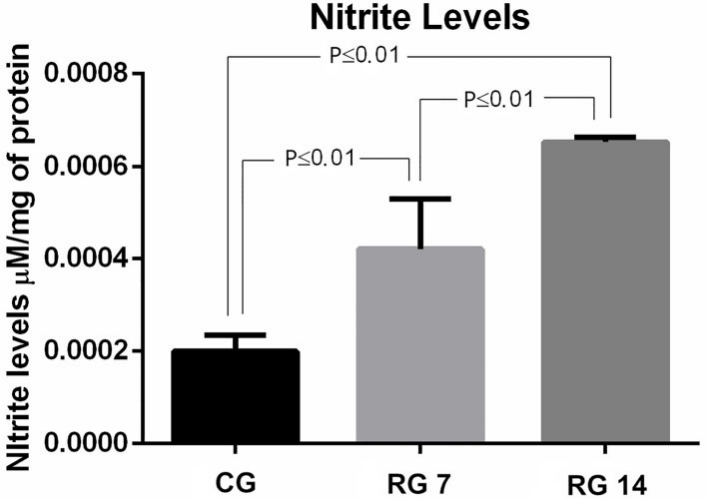
Nitrite quantification in the spinal cord at the 7th and 14th days post-Achilles tendon rupture. The lumbar intumescence was removed to assess nitrite levels in the tissue by the Griess method. Data are reported as means±SD, n=5-6/group (ANOVA followed by Tukey's *post hoc* comparisons). CG: control group; RG 7: rupture group analyzed on the 7th day after tenotomy; RG 14: rupture group analyzed 14th day after tenotomy.

No staining for COX-2 was found in L5 spinal nerve of tenotomized animals on the 7th day post-injury. However, Achilles tendon rupture induced intense expression of COX-2 in the spinal cord on the 14th day post-rupture (Figure 7). Similarly, the expression of NOS-2 in the L5 spinal nerve was only detected on the 14th day post-rupture (Figure 8). The immunoreactivity of these inflammatory mediators (NOS-2 and COX-2) were concentrated on dorsal horns of the spinal cord and ipsilateral side of the ruptured tendon. Nitrite production in the L5 spinal cord was also evaluated in control and ruptured groups. Our data revealed that ruptured animals presented increased levels of nitrite in the L5 spinal cord compared with control on the 7th and 14th days post-Achilles tendon rupture (Figure 9).

## Discussion

The current study demonstrated for the first time that total rupture of Achilles tendon induced microglial activation and inflammatory response in the L5 spinal cord. Although it is widely described that total rupture of Achilles tendon promotes a painful recovery process in injured patients, there are few data in the literature describing the effect of tendon injury on the CNS (21,30,31). As previously described, rupture of the Achilles tendon triggers *in loco* activation of inflammatory response characterized by hypercellularity, intense enervation, and local angiogenesis (30,31).

The L5 spinal cord segment is the region of the CNS that receives input from the Achilles tendon-muscle complex (21). The significant time-dependent astrogliosis and microglial activation observed in this region suggested that Achilles tendon rupture elicits activation of neuroinflammatory response in the spinal cord at the L5 segment. In addition, a significant decrease in PWT values in the ipsilateral paw was observed, which was sustained until the 14th day post-surgery. The hallmarks of neuroinflammation are the activation and infiltration of leukocytes, activation of glial cells, and increased production of inflammatory mediators (32,33). The interactions between inflammation and pain are bidirectional; nociceptive sensory neurons not only respond to immune signals, but also directly modulate inflammation (33). In this way, although further studies are necessary to ratify our hypothesis, the data presented in the current study suggest that the inflammatory response in the L5 segment elicited by tendon rupture could be an important regulator of pain.

Numerous non-neuronal cell types influence pain sensation, including immune, epithelial, mesenchymal, and glial cells (32). It is well documented that microglial activation represents an important response to neuronal injuries (34,35). Our findings are in accordance with previous studies that have described activation of microglial cells in damaged spinal cord of humans and in animal models of spinal nerve injury (25,34).

Data presented in our study suggested that astrocytes could be involved in the maintenance but not in the development of pain, this function being attributed to microglia. In fact, some authors have hypothesized that nerve and spinal cord injuries trigger an initial response of microglia that is followed by astrocytic reactivity. However, future studies are necessary to confirm this hypothesis. In a comparative study utilizing two models of injury, Romero-Sandoval et al. (36) (paw incision and L5 nerve injury) showed that activation of glial cells differs according to the stimulus time, suggesting that the onset and intensity of IBA-1 and GFAP expression are related to the cause of the primary lesion. All our immunostaining results showed greater immunoreactivity in the ventral horns than in the dorsal horns, and we attributed this result to the fact that this is the spinal cord region responsible for the conduction and processing of nociceptive stimuli, which leads to the spinothalamic tract (21). Our data together with those findings indicated the dynamic and plastic nature of glial cells under pathological conditions, and that glial reactivity may demonstrate distinct temporal patterns of expression, depending on the lesion (36).

Inflammatory response in the L5 spinal cord induced by Achilles tendon rupture was confirmed by *in loco* expression of COX-2 and NOS-2 at the 7th and 14th days post-tendon injury. Immunostaining results were also supported by biochemical findings that showed significant elevation of nitrite levels in the lumbar spinal cord of animals submitted to Achilles tendon rupture. It is widely described in the literature that COX-2 and NOS-2 expressions are intimately related to overproduction of prostaglandin E_2_ (PGE_2_) and NO, which are important inflammatory mediators (37,38). PGE_2_ and NO production have also been demonstrated in different kinds of spinal cord and nerve injuries and previous studies point to a close correlation between these mediators and injury in motor performance (21,37). It is widely accepted that peripheral injury induces central changes that can lead to neuroinflammation in the spinal cord, such as peripheral nerve constriction and sciatic nerve (39). This also occurs with nonneuronal peripheral lesion as in induction of unilateral monoarthritis in rats, where an increase in glial reactivity was observed on both sides of the spinal horns at 1, 3, and 10 days after induction (39). Under similar conditions to our experimental model, the immediate production of NO via NOS-2 and PGE_2_ via COX-2 in the spinal cord was attributed to the early induction of peripheral tissue damage and inflammation (38).

**Figure 10:**
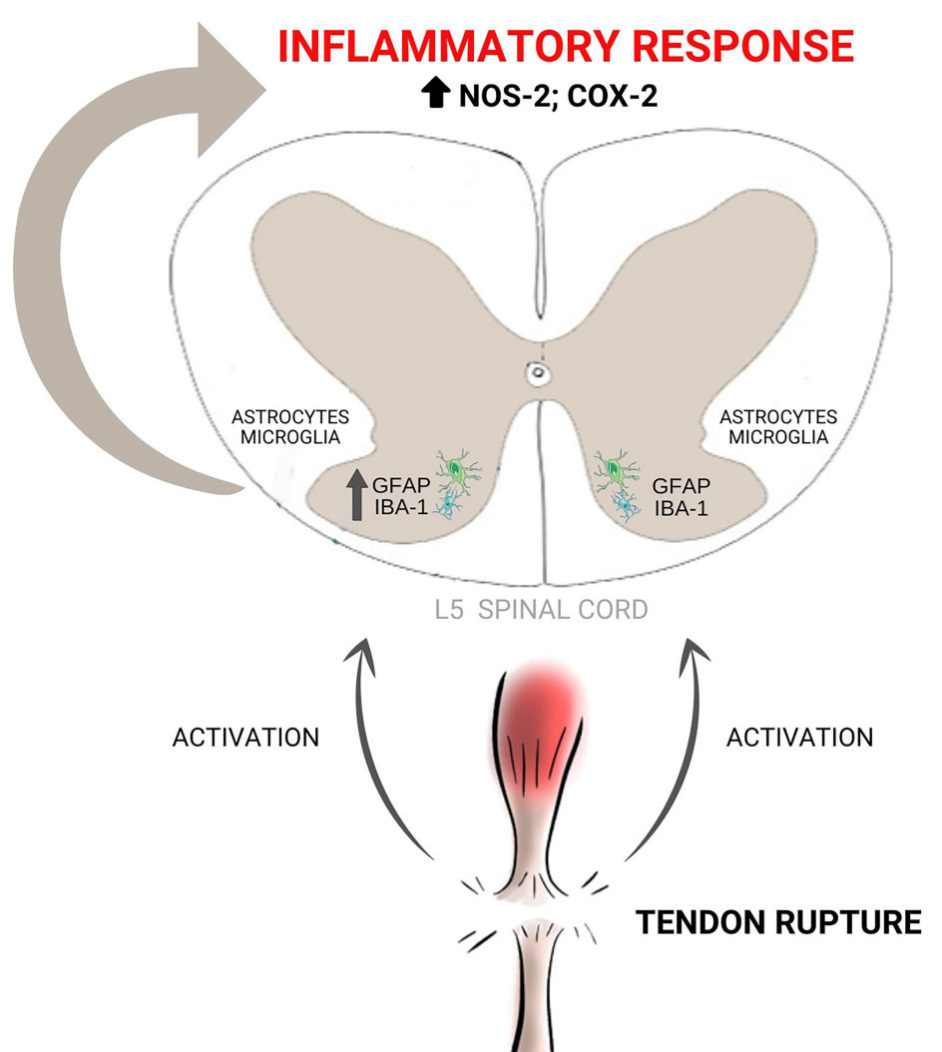
Representative image of inflammatory response in the spinal cord after total Achilles tendon rupture.

In addition, evidence has shown that there is a link between the amount of NO and the severity of painful sensation in patients with chronic pain, who presented a significant increase of nitrite in the cerebrospinal fluid and blood plasma (40). In addition, the central inhibition of COX-2 expression reduces the production of PGE_2_ in the dorsal horns of the spinal cord, with concomitant reduction of mechanical hyperalgesia in a peripheral nerve injury model in rats (24). As shown in Figure 10, our hypothesis is that the Achilles tendon rupture triggers glial activation and production of inflammatory mediators (COX-2 and NO) in dorsal horns of the L5 spinal cord, which favors the generation of neuroinflammation, being a potential physiological mechanism that leads to acute and chronic pain. However, additional studies on neuro-glial interactions are essential to understand the exact mechanism involved in acute and chronic pain induced by Achilles tendon rupture since neurons are also able to express NOS-2 and COX-2.

Taken together our findings demonstrated that total rupture of Achilles tendon directly affects the CNS at the L5 spinal cord segment.
